# ﻿Three new species of *Nautilus* Linnaeus, 1758 (Mollusca, Cephalopoda) from the Coral Sea and South Pacific

**DOI:** 10.3897/zookeys.1143.84427

**Published:** 2023-01-25

**Authors:** Gregory J. Barord, David J. Combosch, Gonzalo Giribet, Neil Landman, Sarah Lemer, Job Veloso, Peter D. Ward

**Affiliations:** 1 Department of Marine Science, Central Campus, Des Moines, Iowa, USA Department of Marine Science, Central Campus Des Moines United States of America; 2 Marine Laboratory, University of Guam, Mangilao, Guam, USA Harvard University Cambridge United States of America; 3 Museum of Comparative Zoology & Department of Organismic and Evolutionary Biology, Harvard University, Cambridge, MA, USA University of Guam Mangilao Guam; 4 Division of Paleontology, American Museum of Natural History, New York, NY, USA American Museum of Natural History New York United States of America; 5 Biology Department, University of Washington, Seattle, WA, USA University of Washington Seattle United States of America

**Keywords:** Conservation, deep-sea, Nautilidae, Nautilus, taxonomy

## Abstract

Nautiloids are a charismatic group of marine molluscs best known for their rich fossil record, but today they are restricted to a handful of species in the family Nautilidae from around the Coral Triangle. Recent genetic work has shown a disconnect between traditional species, originally defined on shell characters, but now with new findings from genetic structure of various *Nautilus* populations. Here, three new species of *Nautilus* from the Coral Sea and South Pacific region are formally named using observations of shell and soft anatomical data augmented by genetic information: *N.samoaensis***sp. nov.** (from American Samoa), *N.vitiensis***sp. nov.** (from Fiji), and *N.vanuatuensis***sp. nov.** (from Vanuatu). The formal naming of these three species is timely considering the new and recently published information on genetic structure, geographic occurrence, and new morphological characters, including color patterns of shell and soft part morphology of hood, and will aid in managing these possibly endangered animals. As recently proposed from genetic analyses, there is a strong geographic component affecting taxonomy, with the new species coming from larger island groups that are separated by at least 200 km of deep water (greater than 800 m) from other *Nautilus* populations and potential habitats. Nautilid shells implode at depths greater than 800 m and depth therefore acts as a biogeographical barrier separating these species. This isolation, coupled with the unique, endemic species in each locale, are important considerations for the conservation management of the extant *Nautilus* species and populations.

## ﻿Introduction

During the mid-19^th^ century period of active study of nautilus taxonomy, little was known about their natural history or even the number of species of extant *Nautilus* Linnaeus, 1758. From the 1980s onward, diverse research concerning the biology and taxonomy of living nautiluses, including aspects of reproduction, inter- and intraspecific variation in shell morphology, distribution, and genetic variation, has shed light on this charismatic clade (Saunders 1981, 1987; Saunders and Landman 1987; Ward 1987; Woodruff et al. 1987; Wray et al. 1995; Bonacum et al. 2011; Kröger et al. 2011; Sinclair et al. 2011; Vandepas et al. 2016; Ward et al. 2016; Combosch et al. 2017; Zhang et al. 2021). This research has profoundly changed *Nautilus* taxonomy and significantly reduced the number of recognized species, often based on single specimens found in Australian waters and otherwise without provenance (Saunders 1987; Ward 1987). It further revealed that there are far more shell characters that can be utilized for taxonomy than previously realized. For example, Ward (1987) cited differences in shell coloration and shell size at maturity, proposing that *Nautilus* populations from Vanuatu and Fiji were sufficiently dissimilar to the type of *N.pompilius* Linnaeus, 1758 to warrant taxonomic differentiation. However, no new species or subspecies were formally defined in that publication.

**Figure 1.**
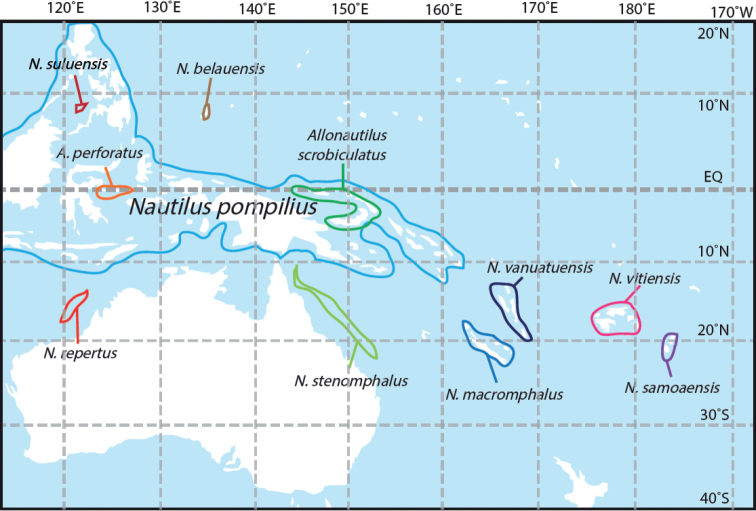
Geographic range map of *Nautilus* and *Allonautilus*.

The number of valid species accepted in the genus *Nautilus* has remained unsettled, even as new genetic information has added to our understanding of the variability within and between *Nautilus* species. Currently, the species that have been described are differentiated based upon their geographic habitat, including the three species described here (Fig. 1).

The three “universally” accepted modern nautilid species of 21^st^ century taxonomists are *Nautiluspompilius* (the type species), *N.macromphalus* G.B. Sowerby II, 1849, and *N.stenomphalus* G.B. Sowerby II, 1849. A fourth, *N.belauensis* Saunders, 1981, has been disputed, and is not currently supported by phylogenetic studies; it does not show significant morphological differences from all other extant species (Vandepas et al. 2016; Combosch et al. 2017; Tajika et al. 2021). It has been placed as a junior synonym of the type *N.pompilius* by Vandepas et al. (2016) but accepted as a valid species elsewhere (e.g., Jereb 2005). Similarly, controversial has been the acceptance of *Nautilusrepertus* Iredale, 1944, which genetic studies have linked with *N.belauensis* (Vandepas et al. 2016; Combosch et al. 2017; Saunders et al. 2017; Tajika et al. 2021), and which is currently considered a *taxon inquirendum* (WoRMS 2021). Additionally, the type designation for *N.pompilius* was recently designated to come from a neotype in Ambon, Indonesia (Nikolaeva et al. 2015), which most significantly brings into question the validity of *N.pompilius* in the Philippines, where a subspecies has already been described by Habe and Okutani (1988) as *N.pompiliussuluensis*. However, recent genome-wide genetic studies did not distinguish this (a single) Indonesian sample from the Philippine *Nautilus* population (Combosch et.al 2017). Future work on the relationship of these two range populations of *Nautilus* would surely improve our understanding of their genetic relationships.

Another significant taxonomic change in *Nautilus* systematics was the definition of the new genus *Allonautilus* (Ward and Saunders 1997). From the time of its first description, *Nautilusscrobiculatus* Lightfoot, 1786 was recognized to be morphologically different from all other nautiluses in shell shape, shell ornament, and shell coloration. However, with the first collection of living specimens of this taxon (Saunders and Davis 1985), significant differences in shell ultrastructure (the presence of a thick periostracum anatomically dissimilar to the homologous layer in all other extant nautilids), and anatomical and color differences of the fleshy hood were demonstrated. Later, dissection of preserved specimens revealed further differences from known species of *Nautilus* in the respiratory and reproductive systems (Ward and Saunders 1997). In consequence, *Nautilusscrobiculatus*, was removed from *Nautilus* and designated instead as the type species of a new genus, *Allonautilus* Ward & Saunders, 1997. Genetic studies (Wray et al. 1995; Sinclair et al. 2007; Bonacum et al. 2011; Sinclair et al. 2011; Vandepas et al. 2016; Combosch et al. 2017) have since confirmed significant genetic differences between *A.scrobiculatus* and the various other species placed in *Nautilus*, further supporting the designation (Bonacum et al. 2011; Vandepas et al. 2016; Combosch et al. 2017; Tajika et al. 2021).

**Figure 2.**
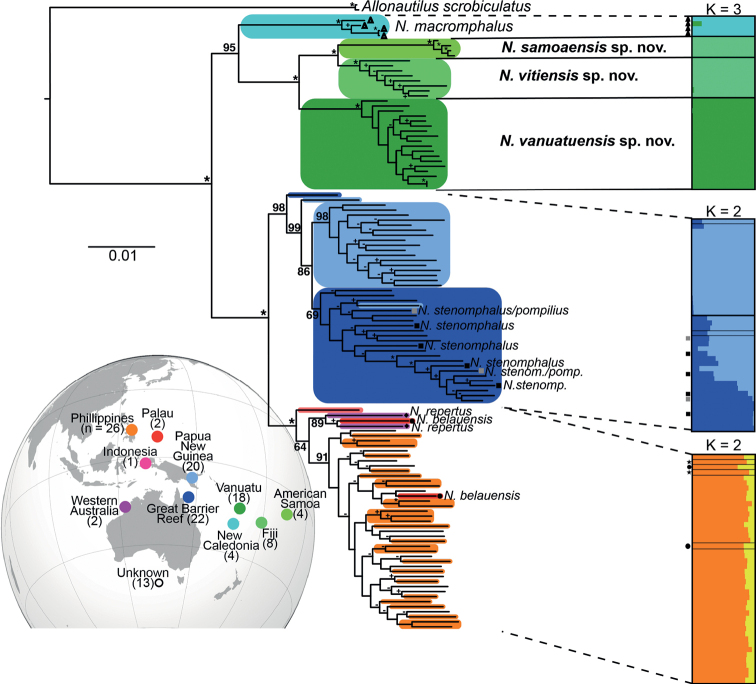
Phylogenetic relationships of nautilids based on maximum-likelihood analysis of 18,595 concatenated SNPs with IQtree as in Combosch et al. (2017). Numbers on nodes indicate ultrafast bootstrap resampling percentages (>50) based on 1000 replicates–asterisks indicate full support (* = 100%). Bootstraps values were replaced on intraclade nodes as follows: “-” for bootstraps values >50 – <90, “+” for bootstrap values ≥90. Colors correspond to geographic populations as indicated in the map on the bottom left. Species other than *Nautiluspompilius* are indicated by name at the tip of the tree. The STRUCTURE plots show the posterior probability for individual assignments of samples to different genetic clusters in clade-specific analyses. Distinct colors indicate different genetic clusters as inferred by STRUCTURE (not populations as on the tree and map).

Early phylogenetic studies based predominantly or exclusively on mitochondrial DNA data indicated major problems with conchological-defined *Nautilus* species and instead identified three geographically distinct clades (Bonacum et al. 2011; Vandepas et al. 2016) and concluded that most *Nautilus* species may not be valid. The subsequent application of genome-wide RAD-Seq data (Combosch et al. 2017) greatly increased the phylogenetic resolution among and within these three major clades and confirmed the presence of genetically distinct subclades among South Pacific Islands in New Caledonia (*N.macromphalus*), Vanuatu (*N.vanuatuensis* sp. nov.), Fiji (*N.vitiensis* sp. nov.), and American Samoa (*N.samoaensis* sp. nov.), of which the three latter species are described further in this paper. Interestingly, some phylogenetic analyses by Combosch et al. (2017) indicated significant genetic differences between Fiji and American Samoa, while a population genetic analysis (STRUCTURE) did not (Fig. 2), which may have been due to the limited sample size from both locations. Ongoing follow-up analyses using expanded datasets and recently generated genome assemblies (Zhang et al. 2021; Huang et al. 2022) will further clarify species boundaries and provide additional population genetic insights into the drivers of differentiation among island populations.

In parallel with the various genetic studies, ecological observations of *Nautilus* populations have been undertaken (Saunders 1984; Dunstan et al. 2011a; Barord et al. 2014; Ward et al. 2016), including understanding of maximum habitat depths imposed by shell implosion of juveniles and adults. Coupled with the lack of planktonic dispersal of newly hatched nautiluses (Ward 1987), this indicated the potential for geographic isolation of populations surrounded by water depth deeper than implosion depths of 800 m and, thus, the potential for allopatric speciation. The behavior of adult nautiluses observed through ultrasonic tracking (Carlson et al. 1984; Ward et al. 1984; Dunstan et al. 2011b; Ward et al. 2016) is consistent with the hypothesis that nautiluses have limited dispersal ability.

The work of Combosch et al. (2017) provided genome-wide evidence consistent with the view that geographic distances and depths readily crossed by invertebrates and vertebrates with planktonic larval stages are effective barriers to migration for nautiluses. Our new mapping of *Nautilus* and *Allonautilus* populations (Fig. 1) with observable morphological differences as well as information from genetic studies suggest that 200 km of deep water (800 m or greater) between separated populations is sufficient to effectively prevent gene flow. These results documenting the genomic structure in *Nautilus* populations living on the isolated archipelagos of American Samoa, Fiji, and Vanuatu, as well as new morphological information presented below, led to the conclusion that the nautiluses at each of these archipelagos are part of distinct populations recognized as new species. While three of these have heretofore been placed in *N.pompilius* (e.g., Ward et al. 1977; Saunders 1987; Ward 1987; Woodruff et al. 1987; Bonacum et al. 2011), in this paper we define each as a new species of *Nautilus*, based on shell characters and hood morphology as well as previously published genome-wide population genetics results.

## ﻿Materials and methods

**Figure 3.**
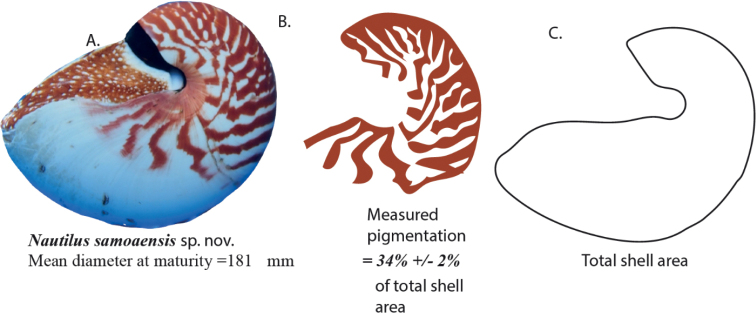
Example of method used to determine percentage of pigmentation on lateral portion of shell in *N.samoaensis* sp. nov.

We use a combination of previously published data, as well as measurements from both museum collections and from newly collected specimens to produce the complete character assemblage data presented here. The combination of morphological characters included: shell coiling descriptors, shell ornament, umbilicus, and hood morphology. One aspect of extant shell morphology that has not been used at the species level is shell decoration, including pattern, pigment hue, and percent of shell covered by pigment. Ward (1987) proposed that the latter (as viewed from the side) was more variable between species than within species and was the basis of the proposal that various species at the time accepted as *N.pompilius* in American Samoa, Fiji, and Vanuatu could be discriminated from the types by their different percentages of pigment cover. Here we use the percent of the shell covered with pigment, as viewed from the side, modified from the methods introduced by Ward (1987). When combined with other characters, pigment cover becomes useful for species level distinction. Specifically, we combine pigment cover with size at maturity. Shell color pattern as a discriminative character was separately presented by Ward (1987), who listed data taken from photographs of captured specimens, and then measured with a manual planimeter. We use essentially the same method here but use the far more discriminating software, ImageJ. In both cases, however, the photographs with the shell striping percentage of a side view of the entire shell was manually obtained using the NIH ImageJ program to separately measure the area of the entire shell, and then measuring that area from a side view that was pigmented, by manually tracing each stripe with the “Irregular circular area” tool. The patterns and the actual area of shell they were compared to (which excluded the area under the black region beneath the hood, and the umbilical region), is shown in Fig. 3.

Additionally, a new character used here for species-level definition and useful for interspecies discrimination is mean shell diameter of mature specimens, as in Ward (1987) and Saunders (1987). All measures presented here come from photographs of either empty shells that are part of museum collections or living animals that were photographed and soon after released. In some cases, photographs and videos were also obtained from *Nautilus* populations in American Samoa, Fiji, and Vanuatu using baited remote underwater video systems (BRUVS) as described by Dunstan et al. (2011a) and Barord et al. (2014). Shell sizes were taken from various sources where size measurements were presented; these sources are listed in the captions of the various diagrams portraying the measured data.

## ﻿Taxonomic section

### ﻿Class Cephalopoda Cuvier, 1795

**Order Ectocochliata Schwartz, 1894**

**Subclass Nautiloidea Agassiz, 1847**

**Family Nautilidae de Blainville, 1825**

#### ﻿Genus *Nautilus* Linnaeus, 1758

##### ﻿ Nautilus pompilius

Taxon classificationAnimaliaEctocochliataNautilidae

Linnaeus, 1758 (type species of Nautilus)

A4119753-6D32-5E5C-A67F-31D43FBE31FE

###### Diagnosis

**(emended from Saunders et al. 2017).** Shell compressed and involute, mature shell size of 140–220 mm mean diameter; 21% pigment coloration; umbilical callus always present. Sexual dimorphism prominent in mature animals: males larger, with a broader aperture than females. Growth lines sinuous, with ocular and hyponomic sinuses well developed in mature shells. Shell surface details from smooth to finely reticulate, because of minute parallel, serial scallops on growth lines in some isolated populations and species. Shell coloration variable, with brown, reddish- to purple-brown, irregular single and bifurcating stripes lacking on the body chamber at maturity. Two discrete color morphs found: stripes continue from venter to umbilicus, and stripes stop at mid flank, with a prominent white patch, sometimes with pale tan color, surrounding umbilicus. Hood covered with flat, white, warty protuberances, with two prominent white ridges extending down midline of hood. Anatomical aspects of the type species recently detailed by Shigeno et al. (2010).

###### Discussion.

A key note from the recent work of Saunders et al. (2017) was dedicated to coloration. In that paper, the explicit statement of there being two color-pattern morphs as a character defining *N.pompilius* was made. While this is found in the types of *N.pompilius* from Ambon, as well as in the new species we define here from Fiji, it is not known from *N.belauensis*, *N.macromphalus*, or *N.vanuatuensis*, whereas color stripes to the umbilicus are not known from *N.repertus* or *N.stenomphalus*. We thus feel justified in proposing this as but one more useful, species-level character when combined with other characters. Here we have used photographs published by Saunders et al. (2017) as means of measuring the percent coloration of the newly defined type species of *N.pompilius*.

##### ﻿ Nautilus vitiensis sp. nov.

Taxon classificationAnimaliaEctocochliataNautilidae

DBC57B5C-9204-50EF-9748-DC5A6738101F

https://zoobank.org/3AEB74DA-AC84-41CC-A622-0EA7AC3D41EE

Fig. 4

###### Type material.

**Figure 4.**
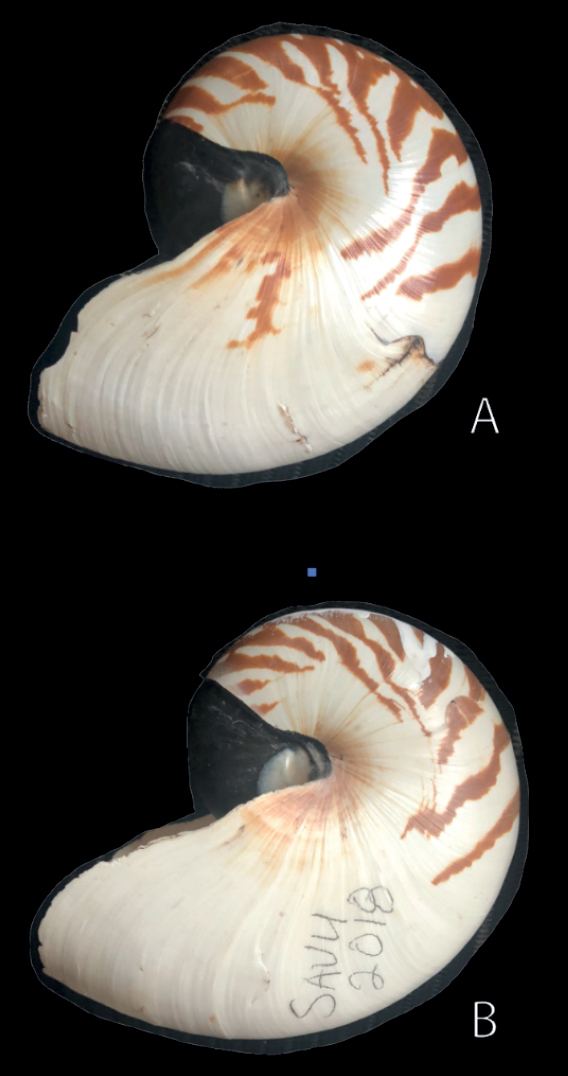
Lateral view of *Nautilusvitiensis* sp. nov., holotype USPMS #12232.

***Holotype***: accessioned at University of South Pacific Marine Station (USPMS) #12232 (Fig. 4). Collected from Suva Harbour, Fiji, 18°07'10.2"S, 178°24'45.0"E, at depths between 250 and 300 m on 30 Jul. 2018.

###### Diagnosis.

The following characteristics distinguish *Nautilusvitiensis* sp. nov. from other species within the genus *Nautilus*: 15–30% pigment coloration on shell, more than in *N.pompilius* and less than in the other species described here; two color pattern morphs present, with both full (stripes from venter to umbilicus) and “umbilical white patch” variety, where stripes flowing down from venter stop short of umbilical region; shell color patterns composed of simple stripes beginning at venter and then extending down the side of shell; these are large and unbranched and are among the simplest of all nautilus shell coloration patterns. The largest specimens of the new species are smaller than the smallest mature *N.pompilius*, and this species is in general smaller than the other two new species described here, but there is certain overlap.

###### Description.

Nautiliconic, shell with umbilical plug, whorl higher than broad at maturity. Periostracum entirely absent in mature and near mature specimens; shell surface ornamented with growth lines parallel to apertural shape; no cross-hatching or ornament perpendicular to growth lines; low rugae. Hood morphology consists of low, elliptical white protuberances barely projecting above hood surface on either side of two long, raised, parallel white stripes running centrally down hood from shell whorl to aperture. White protuberances found between stripes on the entire central section (see hood details on Suppl. material 1: video 1; Fig. 7). Mean diameter of adult shell 149.3 mm, s.d. 7.548 (see range of measured specimens in Table 1).

**Table 1.** Descriptive statistics of mature shell diameters measured of each of the three new species described as well as *N.pompilius* from the type locality at Ambon, Indonesia.

	*N.pompilius*	*N.vitiensis* sp. nov.	*N.samoaensis* sp. nov.	*N.vanuatuensis* sp. nov.
*N*	28	35	10	18
Minimum (mm)	187	137	162	150
Maximum (mm)	207	165	177	163
Range (mm)	20	28	15	13
Mean	195.6	149.3	181.2	156.6
Std. deviation	5.144	7.548	5.029	5.237
Std. error	0.9721	1.276	1.590	1.234

###### Etymology.

The specific epithet, an adjective, refers to the type locality, the island of Viti Levu, Fiji, where the type specimen plus additional released specimens sampled for genetic work were collected.

###### Habitat and distribution.

*Nautilusvitiensis* sp. nov. inhabits areas along the coast of Viti Levu, Fiji at Suva Harbour and Pacific Harbour. Specimens were collected and filmed (Suppl. material 1: video 1) at depths between 200–400 m (Tajika et al. 2022).

##### ﻿ Nautilus samoaensis sp. nov.

Taxon classificationAnimaliaEctocochliataNautilidae

EF157B27-0FC9-590E-A506-8C5D9BDFE0B6

https://zoobank.org/3919775B-5DB9-410C-9871-BE5118D91784

Fig. 5

###### Type material.

**Figure 5.**
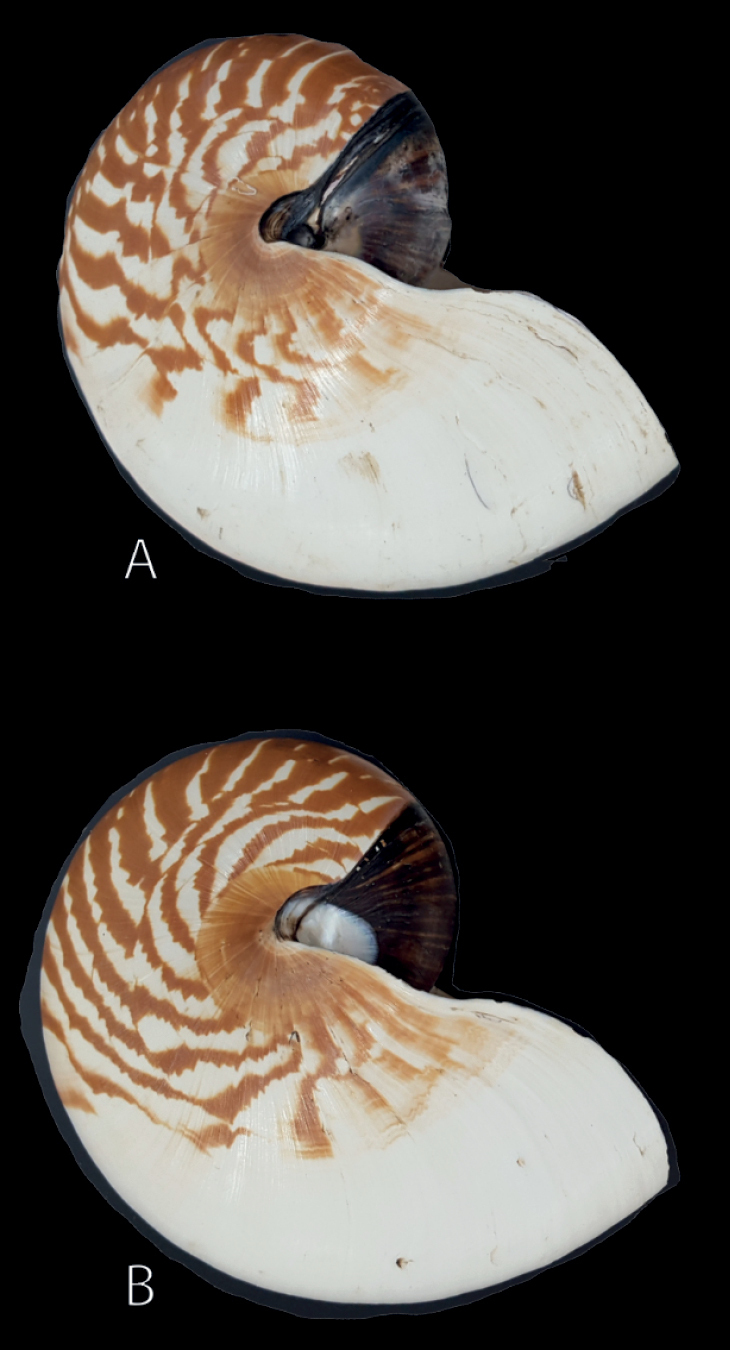
Lateral view of *N.samoaensis* sp. nov. **A** holotype USNM 816658 **B** paratype USNM 816708.

***Holotype***: accessioned at the Natural History Museum, Smithsonian Institution, USNM 816658 (Fig. 5a). Collected in Taema Bank, American Samoa, 14°16'32.27"S, 170°41'12.58"W, between depths of 280 and 310 m on 22 July 1986. ***Paratypes***: accessioned at Natural History Museum, Smithsonian Institution, USNM 816708 (Fig. 5b), same collecting data as holotype. Accessioned at American Museum of Natural History, AMNH 81945, same collecting data as holotype.

###### Diagnosis.

The following characteristics distinguish *Nautilussamoaensis* sp. nov. from other species within the genus *Nautilus*: 32–36% pigment coloration, more than *Nautilusvitiensis* sp. nov. and less than *Nautilusvanuatuensis* sp. nov.; composed of stripes beginning at venter and then curving around in an arc pointing toward aperture as they finally intersect with umbilical region. As noted by Saunders et al. (1989), there is a faint, growth-line sized pattern of annual rings similar in scale and morphology to that found in *Nautilusbelauensis*, but far less marked. Shell color pattern most unique of all *Nautilus* species composed of multiple, branching stripes that have a rearward projection after descending from venter. No other known *Nautilus* species shows this color pattern, and shells are recognized because of this unique coloration pattern, coloration percentage, and shell shape like *N.pompilius*.

###### Description.

Nautiliconic, shell with umbilical plug, whorl higher than broad at maturity. Periostracum entirely absent in mature and near-mature specimens. Shell striping with a series of concentric circles that overlap in a way unique to this species, with a single exception, although this has been referred to as a “zigzag” pattern elsewhere. White protuberances found between stripes and outside of stripes on the entire central section (see hood details in Suppl. material 2: video 2; Fig. 7). Average shell diameter 171.3, s.d. 5.029 (see range of measured specimens in Table 1).

###### Remarks.

A single shell described and illustrated by Saunders et al. (1989) shows an identical pattern to that seen on every observed specimen of *Nautilussamoaensis* sp. nov., but that specimen (USNM 816705) is said to have come from Fiji. However, in the many Fijian nautilus shells with published figures, or examined by us, there has never been another with this pattern, nor has there ever been a shell of this size found in Fiji, so this specimen may have been mislabeled.

The color in freshly caught animals and as viewed underwater has a more magenta hue than is seen in dried shells from this locality. This has not been quantified, however, and as in all other known species, the color changes after the shell dries, red hues are lost, and the shell acquires a uniform brown color.

###### Etymology.

The specific epithet, an adjective, refers to the type locality, American Samoa.

###### Habitat and distribution.

*Nautilussamoaensis* sp. nov. inhabits areas near Pago Pago, American Samoa. Specimens were collected and filmed (Suppl. material 2: video 2) at depths between 200 and 400 m.

##### ﻿ Nautilus vanuatuensis sp. nov.

Taxon classificationAnimaliaEctocochliataNautilidae

3367FB77-78BA-55DF-A6D9-9841CFB5A948

https://zoobank.org/D6847516-191D-43B7-BB36-F9532E8D6D7B

Fig. 6

###### Type material.

**Figure 6.**
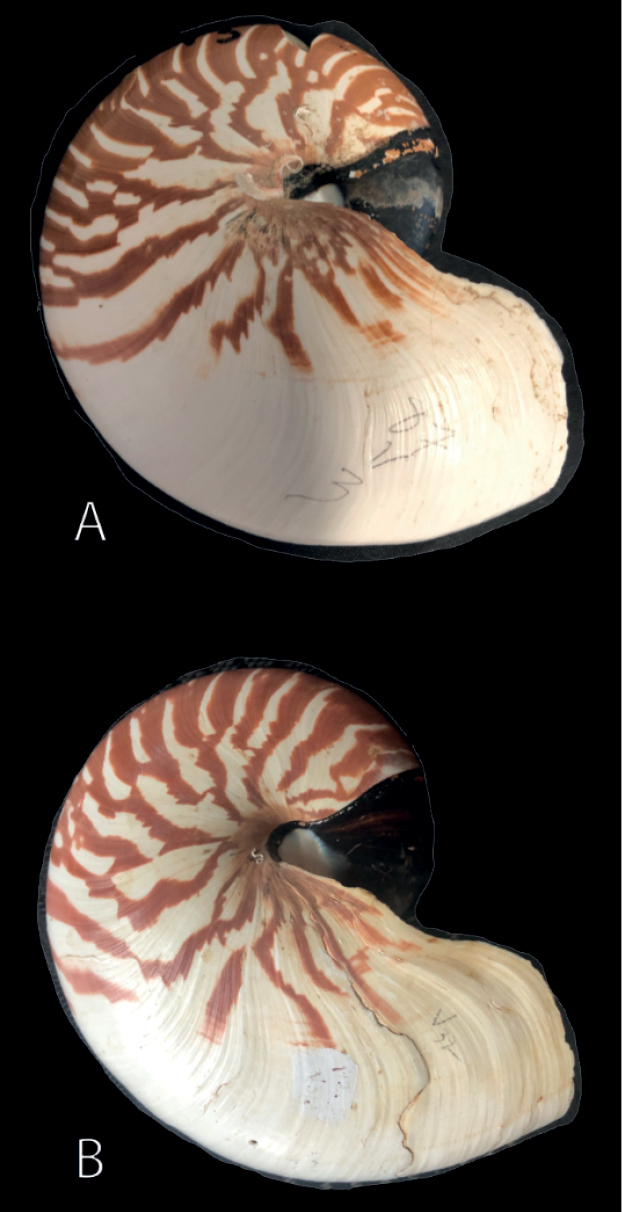
Lateral view of *Nautilusvanuatuensis* sp. nov. **A** holotype AMNH 131861 **B** paratype AMNH 131856.

***Holotype***: accessioned at American Museum of Natural History, AMNH 131861 (Fig. 6a). Collected from Mele Bay, Port Vila, Vanuatu, 17°43'21.61"S, 168°16'01.98"E, at a depth of 185 m on 20 July 2004. ***Paratypes***: accessioned at American Museum of Natural History, AMNH 131856 (Fig. 6b), same collecting data as holotype.

###### Diagnosis.

The following characteristics distinguish *Nautilusvanuatuensis* sp. nov. from other species within the genus *Nautilus*: 40–50% shell coloration, more than any other *Nautilus* species with a plugged umbilicus; pigmentation always composed of stripes extending from venter to umbilicus (no specimens show the “white patch” coloration of *N.pompilius* or *N.vitiensis* sp. nov.). This species is most similar in size, color pattern, and degree of shell covered by pigment to *N.macromphalus*, but the umbilical plug is always missing in the latter species.

###### Description.

**Figure 7.**
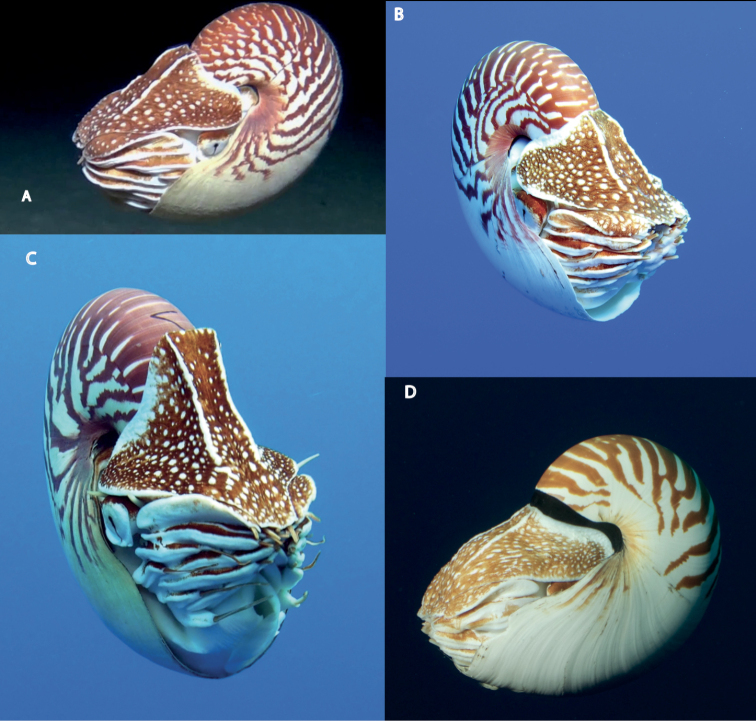
Underwater photos of living *Nautilus***A, B***N.samoaensis* sp. nov. **C***N.vanuatuensis* sp. nov. **D***N.vitiensis* sp. nov.

Nautiliconic, shell with umbilical plug, whorl higher than broad at maturity. Periostracum entirely absent in mature and even near-mature specimens. Shell surface ornamented with growth lines parallel to apertural shape. No cross-hatching or ornament perpendicular to growth lines. Hood morphology consisting of low, elliptical white protuberances barely projecting above hood surface on either side of two long, raised, parallel white stripes running centrally down hood from shell whorl to aperture. White protuberances found between stripes on entire central section; they are low and non-digitate at their terminal ends (see hood details on Suppl. material 3: video 3; Fig. 7). Mean diameter of adult shell is 156.6 mm, s.d. 5.237 (see range of measured specimens in Table 1).

###### Discussion.

*Nautilusvanuatuensis* is virtually identical in size, color pattern, and degree of shell covered by pigment to *N.macromphalus* in New Caledonia, which is the *Nautilus* species geographically closest to Vanuatu. The new species we define here differs by always having an umbilical plug. Both species are ecologically similar in that mature specimens are commonly observed in very shallow water (up to 5 m depth) off Vanuatu and New Caledonia. As species show the similar, high degree of pigmentation, we assume that the shallow-water habitat visitation by both is the reason for their high level of pigmentation.

###### Etymology.

The specific epithet, an adjective, refers to the type locality, Vanuatu, where all the known specimens have been collected.

###### Habitat and distribution.

*Nautilusvanuatuensis* inhabits sites within Mele Bay, Vanuatu. Specimens were collected and filmed (Suppl. material 3: video 3) at depths of 200–400 m.

### ﻿Conservation status

All nautiluses (family Nautilidae) are regulated under appendix II of the Convention on International Trade in Endangered Species (CITES). As of this writing, no species of *Nautilus* or *Allonautilus* has been formally assessed by the International Union for Conservation of Nature Red List.

## ﻿Discussion

The populations of nautiluses within American Samoa, Fiji, and Vanuatu have already been effectively isolated from each other because of warm, surface seawater temperatures, depth implosion limits below 800 m, and their nektobenthic lifestyle, one of endless foraging just above the bottom, and thus within a few meters of the benthic environment at most. Here, we combined morphological characteristics with previously published population genomic results to newly describe each population of *Nautilus* within these archipelagos as a unique species. The characters that showed the most differentiation between the species included multiple color pattern traits (i.e., percent of shell pigmented in matures specimens and striping patterns) and, while not yet quantified, the actual “hue” of pigment in freshly caught specimens.

**Figure 8.**
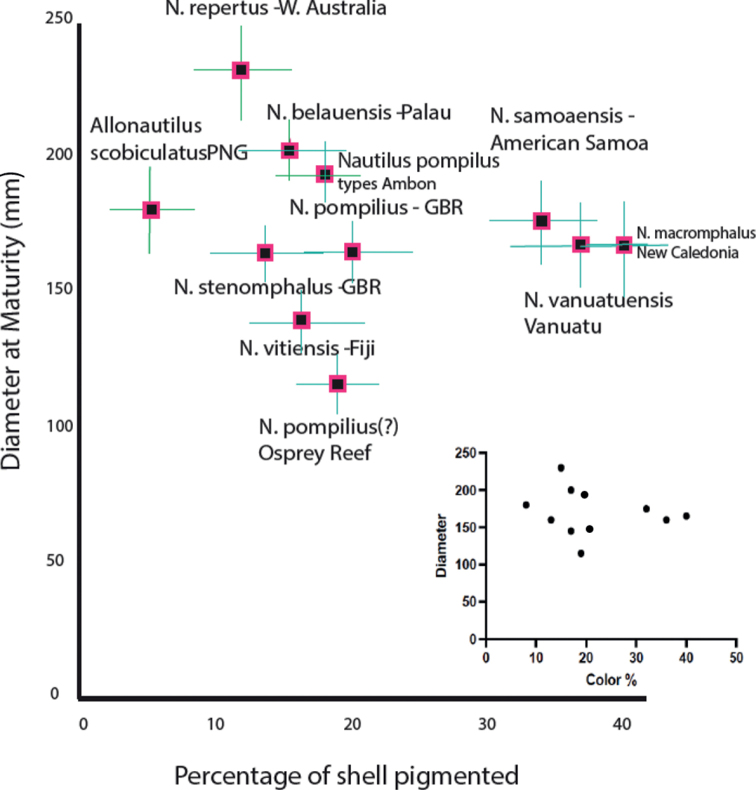
Shell diameter and shell pigmentation relationship of *N.vitiensis* sp. nov., *N.samoaensis* sp. nov., and *N.vanuatuensis* sp. nov. compared against each other as well as other *Nautilus* species and populations.

Fig. 7 shows the shell decoration and hood ornamentation of each of the species described here. Shell size at maturity also has discerning potential among some of the species’ groups (Fig. 8). *Nautilussamoaensis* sp. nov., *N.vitiensis* sp. nov., and *N.vanuatuensis* sp. nov. represent the easternmost range of extant nautilids and provide further validation for the decision to regulate all nautiluses (family Nautilidae) within appendix II of the Convention on International Trade in Endangered Species (CITES) given their morphological and genetic differences.

For comparative purposes, if we include the New Caledonian populations of nautiluses, *N.macromphalus*, with the three new species described here we can draw further inferences on the relationship of distinct species of nautiluses. The strongest morphologic similarity among the four species we designate as endemic to this region is between *N.macromphalus* and what the population assigned to *N.pompilius* from the Vanuatu archipelago, *N.vanuatuensis* sp. nov. They are clearly identified by the umbilical region, although indistinguishable in all other studied characters (Ward 1987). These two species are morphologically similar, as well as being geographically proximate to one another and, as noted above, also share the behavioral trait of commonly swimming at night into depths shallower than inhabited by any other *Nautilus* species. However, some of these traits must be adaptational, as these two species are not each other’s closest relatives (Fig. 2).

The third of the four species living in what we call an “South Pacific” biogeographic region (sensu Combosch et al. 2017), *Nautilussamoaensis* sp. nov. resembles both *N.macromphalus* and *N.vanuatuensis* sp. nov. in size and the relative amount of coloration on the shell but differ markedly in shell color “pattern.” It is sister to *N.vitiensis* sp. nov. and while these two species were ambiguously distinguished in prior genetic work, their reciprocal monophyly and the morphological differences outlined above, lead us to designate them as distinct species here.

Individuals of *N.vitiensis* sp. nov. in Fiji have never been seen at night, and the exceptionally low amount of pigmentation in this species may be due to an overall, deeper-water habitat, as discussed above. In any event, *N.vitiensis* sp. nov. has among the lowest degree of shell coloration of all currently defined species. New analyses of previously published shell formation temperatures of oxygen isotopes from shell and septal samples (Tajika et al. 2022) are consistent with the growth of juvenile species at depths below 300 m; these data, however, tell us nothing about habitation depths of the non-growing mature specimens.

Compared to *N.macromphalus* and *N.vanuatuensis* sp. nov., *N.samoaensis* sp. nov. has a unique species-level character: it has the most striking and readily identifiable ornamental pattern of any known *Nautilus* or *Allonautilus* species. Specimens captured with baited traps and observed through BRUVS observations made in 2013 at depths of 250–350 m all show coloration patterns on the middle portion of the phragmocone, which is the rare (for *Nautilus*), single character trait that identifies the species: a complex change in stripe direction (compared to striping found in any other *Nautilus*). Different as it is from the Vanuatu–New Caledonian species couplet, *N.samoaensis* sp. nov. still remains closer in observable shell characteristics to those two South Pacific species than it does to the geographically closer nautiluses of Fiji. The latter are smaller, and their shells bear a significantly different ornamentation than those of the other three species identified within this biogeographic region.

These data may be skewed, as all sampled populations of nautiluses report that males vastly outnumber females in traps, which is related to size at maturity. Nevertheless, the size at maturity remains a powerful character, and correlates well with genetic data. In fact, as we show here, the currently accepted and newly proposed species herein, with the sole exception of *N.pompilius*, can be statistically separated using a combination of the characters of mature shell size, percentage of shell covered by pigment, the morphology of the shell umbilical region, the morphology of the shell surface at a growth line scale, the thickness of the periostracum (which is dependent on shell surface morphology), and the morphology and color of the hood. Undoubtedly, other characters will be discovered when detailed dissection of the accepted species is finally attempted, including comparing the morphology of the radula and jaws. Nevertheless, even with the characters at hand, we show here that populations known to be separated by at least 200 km of deep water maintain significant genetic differences as well as measurable morphological differences, which is consistent with our conclusion that the populations of American Samoa, Fiji, and Vanuatu merit elevation to separate species. The use of morphological characters, shell pattern, and, to a lesser extent, shell size, have additional power when regulating and enforcing current trade regulations for all nautiluses. The fact that we were able to combine the morphology and genetics to differentiate these species provides a foundation for managers and other officials to begin to efficiently identify distinct species of nautilus shells that may come through as trade products.

## ﻿Conclusions

The three species, *N.vitiensis*, *N.samoaensis*, and *N.vanuatuensis* represent populations of nautiluses on the easternmost edge of the overall habitat range of *Nautilus*. The designation of these three populations as distinct species provides insight into evolutionary radiation of the genus and clarification for future conservation practices.

## Supplementary Material

XML Treatment for Nautilus pompilius

XML Treatment for Nautilus vitiensis

XML Treatment for Nautilus samoaensis

XML Treatment for Nautilus vanuatuensis

## Supplementary materials

Supplementary material 1 Supplemental video 1 Data type video file Explanation note Baited remote underwater video systems (BRUVS) observations of *Nautilusvitiensis* sp. nov. recorded at approximately 300 m depth in Fiji. https://binary.pensoft.net/file/800686This dataset is made available under the Open Database License (http://opendatacommons.org/licenses/odbl/1.0/). The Open Database License (ODbL) is a license agreement intended to allow users to freely share, modify, and use this Dataset while maintaining this same freedom for others, provided that the original source and author(s) are credited.Gregory J. Barord, David J. Combosch, Gonzalo Giribet, Neil Landman, Sarah Lemer, Job Veloso, Peter D. Ward

Supplementary material 2 Supplemental video 2 Data type video file Explanation note Baited remote underwater video systems (BRUVS) observations of *Nautilussamoaensis* sp. nov. recorded at approximately 300 m depth in American Samoa. https://binary.pensoft.net/file/800687This dataset is made available under the Open Database License (http://opendatacommons.org/licenses/odbl/1.0/). The Open Database License (ODbL) is a license agreement intended to allow users to freely share, modify, and use this Dataset while maintaining this same freedom for others, provided that the original source and author(s) are credited.Gregory J. Barord, David J. Combosch, Gonzalo Giribet, Neil Landman, Sarah Lemer, Job Veloso, Peter D. Ward

Supplementary material 3 Supplemental video 3 Data type video file Explanation note Baited remote underwater video systems (BRUVS) observations of *Nautilusvanuatuensis* sp. nov. recorded at approximately 300 m depth in Vanuatu. https://binary.pensoft.net/file/800688This dataset is made available under the Open Database License (http://opendatacommons.org/licenses/odbl/1.0/). The Open Database License (ODbL) is a license agreement intended to allow users to freely share, modify, and use this Dataset while maintaining this same freedom for others, provided that the original source and author(s) are credited.Gregory J. Barord, David J. Combosch, Gonzalo Giribet, Neil Landman, Sarah Lemer, Job Veloso, Peter D. Ward
